# Mercury Ion Selective Adsorption from Aqueous Solution Using Amino-Functionalized Magnetic Fe_2_O_3_/SiO_2_ Nanocomposite

**DOI:** 10.3390/ma17174254

**Published:** 2024-08-28

**Authors:** Mahmoud M. Youssif, Heba G. El-Attar, Stanisław Małecki, Grzegorz Włoch, Maciej Czapkiewicz, Kamil Kornaus, Marek Wojnicki

**Affiliations:** 1 Faculty of Non-Ferrous Metals, AGH University of Krakow, al. A. Mickewicza 30, 30-059 Krakow, Poland; stanmal@agh.edu.pl (S.M.); gwloch@agh.edu.pl (G.W.); 2 Department of Chemistry, Faculty of Science, Tanta University, Tanta 31527, Egypt; heba.elattar@science.tanta.edu.eg; 3 Faculty of Computer Science, Electronics and Telecommunications, AGH University of Krakow, al. Mickiewicza 30, 30-059 Krakow, Poland; czapkiew@agh.edu.pl; 4 Faculty of Materials Science and Ceramics, Department of Ceramics and Refractory Materials, AGH University of Krakow, al. Mickiewicza 30, 30-059 Krakow, Poland; kornaus@agh.edu.pl

**Keywords:** Amino-functionalization, adsorption capacity, Hg (II) ion, magnetic nanoparticles, removal of heavy metals

## Abstract

This study focuses on the development of new amino-functionalized magnetic Fe_2_O_3_/SiO_2_ nanocomposites with varying silicate shell ratios (1:0.5, 1:1, and 1:2) for the efficient elimination of Hg^2+^ ions found in solutions. The Fe_2_O_3_/SiO_2_–NH_2_ adsorbents were characterized for their structural, surface, and magnetic properties using various techniques, including Fourier transform infrared spectrum (FT-IR), powder X-ray diffraction (XRD), scanning electron microscopy (SEM), Braunauer–Emmett–Teller (BET), thermogravimetric analysis (TGA), zeta-potential, and particle size measurement. We investigated the adsorption circumstances, such as pH, dosage of the adsorbent, and duration of adsorption. The pH value that yielded the best results was determined to be 5.0. The Fe_2_O_3_/SiO_2_–NH_2_ adsorbent with a silicate ratio of (1:2) exhibited the largest amount of adsorption capacity of 152.03 mg g^−1^. This can be attributed to its significantly large specific surface area of 100.1 m^2^ g^−1^, which surpasses that of other adsorbents. The adsorbent with amino functionalization demonstrated a strong affinity for Hg^2+^ ions due to the chemical interactions between the metal ions and the amino groups on the surface. The analysis of adsorption kinetics demonstrated that the adsorption outcomes adhere to the pseudo-second-order kinetic model. The study of adsorption isotherms revealed that the adsorption followed the Langmuir model, indicating that the adsorption of Hg^2+^ ions with the adsorbent occurred as a monomolecular layer adsorption process. Furthermore, the thermodynamic analyses revealed that the adsorption of Hg^2+^ ions using the adsorbent was characterized by a spontaneous and endothermic process. Additionally, the adsorbent has the ability to selectively extract mercury ions from a complex mixture of ions. The Fe_2_O_3_/SiO_2_–NH_2_ nanocomposite, which is loaded with metal, can be easily recovered from a water solution due to its magnetic properties. Moreover, it can be regenerated effortlessly through acid treatment. This study highlights the potential use of amino-functionalized Fe_2_O_3_/SiO_2_ magnetic nanoparticles as a highly efficient, reusable adsorbent for the removal of mercury ions from contaminated wastewater.

## 1. Introduction

The environment’s concentration of heavy metal ions has been rising over the past ten years. It is imperative to address the harmful metal ions causing water pollution, as it is a grave matter [1,2]. Living organisms can suffer greatly when exposed to polluted water. Mercury, cadmium, lead, nickel, copper, arsenic, and chromium are some of these heavy metals. They can also alter the water’s chemical and physical nature [3,4,5]. The main factor causing hazardous heavy metals to be released into the environment is rapid industrialization. Heavy metal ion contaminations are mostly caused by mining, battery, textile, petroleum refining, and paint manufacture among other industries [6,7]. One of the most prevalent and bio-accumulative contaminants is known to be mercury (Hg). The Environmental Protection Agency (EPA) states that the highest permitted level of mercury is 0.001 mg L^−1^, but the WHO has placed it at 0.002 mg L^−1^ [8,9]. Mercury (Hg) is considered one of the most dangerous pollutants that have created concern worldwide because of its toxicity, which can lead to mental issues, gastrointestinal disorders, discomfort in the muscles, chronic weariness, eyesight problems, and susceptibility to fungal infections [10]. Consequently, the elimination of heavy metals from wastewater is turning into a crucial problem. Many methods have been used to remove heavy metals from aqueous solutions, including flocculation, ion exchange, adsorption, chemical precipitation, electrolysis, and filtering [11,12,13]. Adsorption is extensively used due to its great effectiveness, simplicity of usage, economic consideration, and cost-effectiveness [14,15]. Moreover, the adsorption process does not create any hazardous materials or have any negative effects [16].

Adsorbent materials that have been evaluated and investigated for the potential use of hazardous heavy metal ion adsorption include activated carbon, zeolites, polymers, and biomaterials [17,18,19,20]. Since the adsorption performance is primarily reliant on the adsorbent characteristics, there is now a growing global interest in creating efficient adsorbents for water treatment applications. Thus, a significant number of researchers are focused on creating adsorbents that are more effective. Novel adsorbents based on nanoparticles have been created with the goal of eradicating heavy metal ions from wastewater with enhanced adsorption performance [21,22,23]. It has been observed that magnetic metal oxide nanoparticles are economical, highly effective, and efficient adsorbents. Moreover, they recover easily by easily separating in a magnetic field [24,25,26,27]. Metal oxide nanocomposites’ large surface area and smaller particle sizes allow them to improve the adsorption efficiency of ions well. Because metal oxide nanocomposites have special morphological characteristics, they would thus be particularly successful in the adsorption process. Recently, silica was used to cover maghemite to generate a magnetic nano adsorbent for the removal of metal ions from aqueous solutions. Furthermore, because amino groups have a high metal complexing ability, the amino-functionalized magnetic materials showed exceptional ability to eliminate a broad variety of heavy metal ions from aqueous solutions, including Hg^2+^, Cu^2+^, Co^2+^, Ni^2+^, Zn^2+^, Pb^2+^, and Cd^2+^ ions [28,29,30,31]. The stabilization and effectiveness of magnetic nanoparticles (MNPs) for the elimination of metal ions are improved by surface functionalization of magnetic nanomaterials, as demonstrated by several investigations [32]. Amino-functionalization MNPs have superior adsorption efficacy for Hg^2+^ ions compared to other modifications since Hg^2+^ ions have a considerable affinity for binding with amino-containing groups [33].

In this study, the surfaces of Fe_2_O_3_/SiO_2_ nanocomposites with different distinct ratios of silicate (1:0.5, 1:1, and 1:2) were covalently grafted with amino groups to create unique magnetic nanoadsorbents. Subsequently, using batch adsorption examinations, the adsorption characteristics of the Fe_2_O_3_/SiO_2_–NH_2_ adsorbent were examined for the elimination of toxic mercury ions for water purification under various conditions. Furthermore, the study looked at how the adsorption–desorption process and the coexistence of ions affected the adsorption capacity. Lastly, an examination of the adsorption process was conducted using the results of the kinetic and isotherm studies. We focused on the adsorption capacity, material durability, and regeneration of the magnetic adsorbents towards Hg^2+^ ions in an aqueous solution.

## 2. Experimental

### 2.1. Chemical Reagents

Tetraethyl orthosilicate (TEOS), anhydrous sodium acetate (NaAC), ethylenediamine, polyvinylpyrrolidone (PVP), ammonium hydroxide (25%), and 3-aminopropyltriethoxysilane were obtained from Sigma-Aldrich. Sodium hydroxide was obtained from Avantor Performance Materials, Gliwice, Poland; hydrochloric and nitric acids were obtained from Chemland Materials, Stargard, Poland, as well as HPLC-grade ethanol and toluene. Analytical-grade metal salts, including FeCl_3_∙6H_2_O, Mg(ClO_4_)_2_, ZnCl_2_, CuCl_2_∙2H_2_O, NiCl_2_∙2H_2_O, CdCl_2_∙2H_2_O, and HgCl_2_, were employed to prepare stock solutions containing 1000 mg L^−1^ of each one. All analytical-grade chemicals and reagents are used without additional purification.

### 2.2. Instrumentation

X-ray diffraction (XRD) was conducted using a Rigaku MiniFlex II instrument manufactured in Tokyo, Japan to analyze the crystalline arrangement of nanoparticles and ascertain the grain size. Scanning electron microscopy (SEM) was conducted using Hitachi SU-70 (Tokyo, Japan) to analyze the nanocomposites’ morphology, size, and size distribution. An energy-dispersive X-ray spectroscope (EDS) (Waltham, MA, USA) was connected to the scanning electron microscope, allowing it to perform elemental analysis. It operates at an accelerating voltage of 15.00 keV. The Fourier transform infrared (FT-IR) spectra were recorded using Nicolet (Waltham, MA, USA). The FT-IR spectroscopy was performed using the transmission method. The samples were ground in an agate mortar with 0.2 g of FT-IR grade (≥99%) KBr (Sigma-Aldrich, St. Louis, MO, USA). The samples were manufactured utilizing a punch with a diameter of 10 mm and a hydraulic press that can produce pressures of up to 10 MPa. Measurements of the BET surface area were conducted using a Micromeritics ASAP 2010 instrument. The samples underwent degassing at a temperature of 200 °C to a vacuum of 4 µmHg for 24 h. The measurement begins after a vacuum of 10 µmHg is reached in the measuring cell. The initial measurement is conducted within a pressure range of 40 to 50 mm of mercury (mmHg). The temperature of the measurement cell was set to 77.35 K, and each sample was measured five times with progressively higher relative pressure (the interval between measurements was 120 min). The magnetization and hysteresis loop were measured at room temperature using a vibrating sample magnetometer (VSM, LDJ 9600, LDJ Electronics Company of USA, Ventura, CA, USA). A Malvern Zeta Sizer Nano ZS (Malvern Instrument Ltd. Malvern, UK) was used to examine the particle size distribution and zeta potential of the colloidal suspensions. The thermogravimetric analysis (DSC–TGA) was determined using TA Instruments V4.3A (TA instrument, New Castle, DE, USA). The temperature was raised from 25 °C to 800 °C, with an increment rate of 10 °C/min. The materials underwent thermal studies in an argon environment, with a gas flow rate of 100 mL/min, unless specified differently. The remaining Hg^2+^ ions in adsorption/desorption tests were measured using MP-AES (Agilent 4200, Santa Clara, CA, USA).

### 2.3. Synthesis of Fe_2_O_3_ Nanoparticles

To synthesize Fe_2_O_3_ nanoparticles, mix 1 g of polyvinylpyrrolidone (PVP) and 2 g of FeCl_3_∙6H_2_O in 30 mL of water and stir until a clear solution forms. The solution was then supplemented with 2 g of anhydrous sodium acetate and 7 mL of ethylenediamine. The mixture was then placed in an autoclave with a hermetically sealed lid and heated to 200 °C for ten hours. The Fe_2_O_3_ nanoparticles were carefully cleaned with water after collecting and then dried in a 60 °C oven [34].

### 2.4. Synthesis of Fe_2_O_3_/SiO_2_ Nanocomposites with Different Thickness of SiO_2_

**Figure 1:**
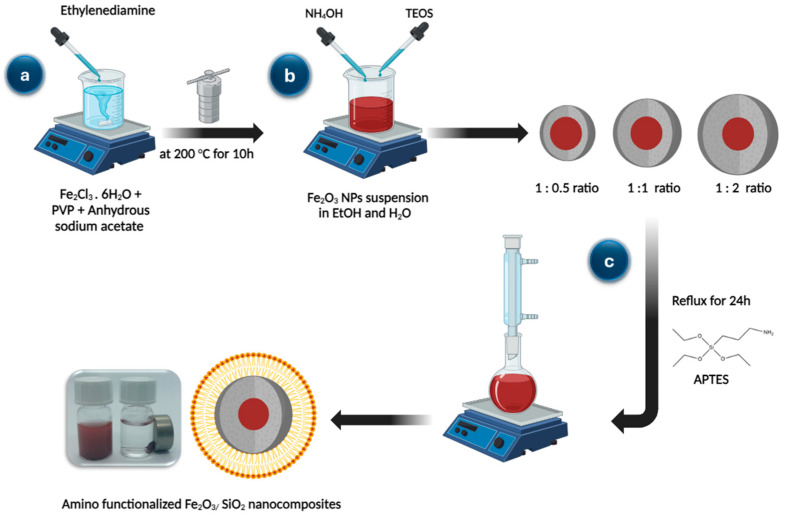
Synthesis of (**a**) Fe_2_O_3_ nanoparticles, (**b**) Fe_2_O_3_*/*SiO_2_ with different ratio (1:0.5, 1:1, and 1:2), and (**c**) amino-functionalization of Fe_2_O_3_*/*SiO_2_ nanocomposites.

In total, 2 g of Fe_2_O_3_ nanoparticles were mixed with 180 mL of ethanol and 50 mL of water and sonicated for 30 min. The solution was then gradually supplemented with 10 mL of 25% ammonium hydroxide dropwise. The mixture was left to stir for 6 h at a temperature of 25 °C. Centrifugation was used to gather the produced Fe_2_O_3_/SiO_2_, which was then washed serval times using water and ethanol. After that, the composite was allowed to dry in a 60 °C oven and then kept for subsequent use in a desiccator Figure 1b [35]. Varying amounts of tetraethyl orthosilicate (TEOS) with Fe_2_O_3_ nanoparticles were used in this study. Fe_2_O_3_:TEOS ratios were 1:0.5, 1:1, and 1:2 (using 1, 2, and 4 mL of TEOS for every 2 g of Fe_2_O_3_).

### 2.5. Amino-Functionalization of Fe_2_O_3_/SiO_2_ Nanocomposites

The synthesized Fe_2_O_3_/SiO_2_ nanocomposites can undergo chemical functionalization on both their interior and exterior surfaces using various ways. This is accomplished by linking a functional group using either a post-synthesis or a one-step approach. As a result, the post-synthesis technique has been extensively used to achieve significant loading of functional groups [36]. The post-synthesis technique involves grafting the functional group into the Fe_2_O_3_/SiO_2_ nanocomposites that have been produced Figure 1c. The Fe_2_O_3_/SiO_2_ nanocomposites were amino-functionalized using the following procedure: A solution of 1.8 g of 3-aminopropyltriethoxysilane dissolved in 150 mL of dry toluene was mixed with 1 g of Fe_2_O_3_/SiO_2_ nanocomposites. The mixture was then allowed to reflux for a whole day. To get rid of any unreacted silane that remained, the solid was filtered and repeatedly cleaned with ethanol and toluene. Following that, it was vacuum-dried at 50 °C [37]. 

### 2.6. Batch Adsorption Experiments

The adsorption performance of the Fe_2_O_3_, Fe_2_O_3_/SiO_2_, and Fe_2_O_3_/SiO_2_–NH_2_ nanocomposites for Hg^2+^ ions was investigated using a batch approach. The experiments were carried out in the following manner: Stock solutions containing Hg^2+^ ions at a concentration of 1000 mg L^−1^ were prepared using deionized water. This solution was then diluted to obtain final concentrations ranging from 10 to 50 mg L^−1^. The pH of the solution was first adjusted within a range of 1 to 9 using 0.1M NaOH and 0.1M HCl, with the assistance of a universal buffer solution. Typically, 10 mg of each magnetic nanocomposite was added into a solution containing 50 ml of metal ions with a pH of 5. The mixture was then subjected to ultra-sonication for a few minutes and transferred to a water thermostatic shaker set at an agitation speed of 140 rpm and a temperature of 30 °C. The time at which the magnetic nanocomposite was introduced into the reaction mixture was recorded. The kinetics measurements started promptly with the addition of the nanocomposite, and the solution samples were collected at various time intervals (ranging from 10 to 180 min). The impact of temperature was investigated by subjecting the sample to agitation within the temperature range of 298 to 318 K. Subsequently, the magnetic adsorbent was extracted from the solution. The concentration of Hg^2+^ ions in the supernatant was determined using an Atomic Emission Spectrometer (Agilent 4200) known as (MP-AES) by using standard solutions of Hg^2+^ ions (0.5 to 80 mg L^−1^). Equation (1) was used to estimate the removal efficiency (R%), whereas Equations (2) and (3) were used to quantify the amount of metal ions adsorbed at definite time and equilibrium.

(1) $$R\%=\frac{C_{O}-C_{t}}{C_{o}}\cdot 100\%$$

(2) $$q_{t=\frac{C_{O}-C_{t}}{C_{o}}\cdot \frac{V}{m}}$$

(3) $$q_{e=\frac{C_{O}-C_{e}}{C_{o}}\cdot \frac{V}{m}}$$

The symbol *C_o_* represents the initial concentration of metal ions; *C_e_* represents the equilibrium concentration; and *C*_t_ represents the concentration of metal ions at a certain time t. *R* (%) is the removal efficiency, and q_t_ and *q_e_* (mg∙g^−1^) represent the adsorption capabilities of the Fe_2_O_3_/SiO_2_–NH_2_ nanocomposites at a certain time *t* (in minutes) and at equilibrium. The volume of Hg^2+^ solution is expressed as *V* (L), and the mass of adsorbent is described as *m* (g).

### 2.7. Desorption and Regeneration Studies

To evaluate the possibility of recycling, the most effective magnetic nanoadsorbent for the adsorption of Hg^2+^ ions was selected. The process of regenerating Hg-loaded Fe_2_O_3_/SiO_2_–NH_2_ (1:2 ratio) was achieved by separating it from the medium via magnetic separation. To explore the nanocomposite’s potential for reuse for the adsorption of metal ions, the nanocomposite was mixed with 25 mL of a solution containing 0.1 M HNO_3_ and 0.1 M HCl. The mixture was then exposed to ultrasonication for 10 min and stirred for 12 h at a temperature of 25 °C. This process aimed to remove heavy metal ions from the adsorbent. Afterward, the nanocomposite was magnetically separated from the solution and then followed by several washes with distilled water. Subsequently, the Fe_2_O_3_/SiO_2_–NH_2_ (1:2 ratio) nanocomposite that had been recycled was used in further adsorption experiments, maintaining the same conditions. This process was replicated for five cycles.

## 3. Results and Discussion

### 3.1. Characterization of Adsorbent

#### 3.1.1. FT-IR Study

**Figure 2:**
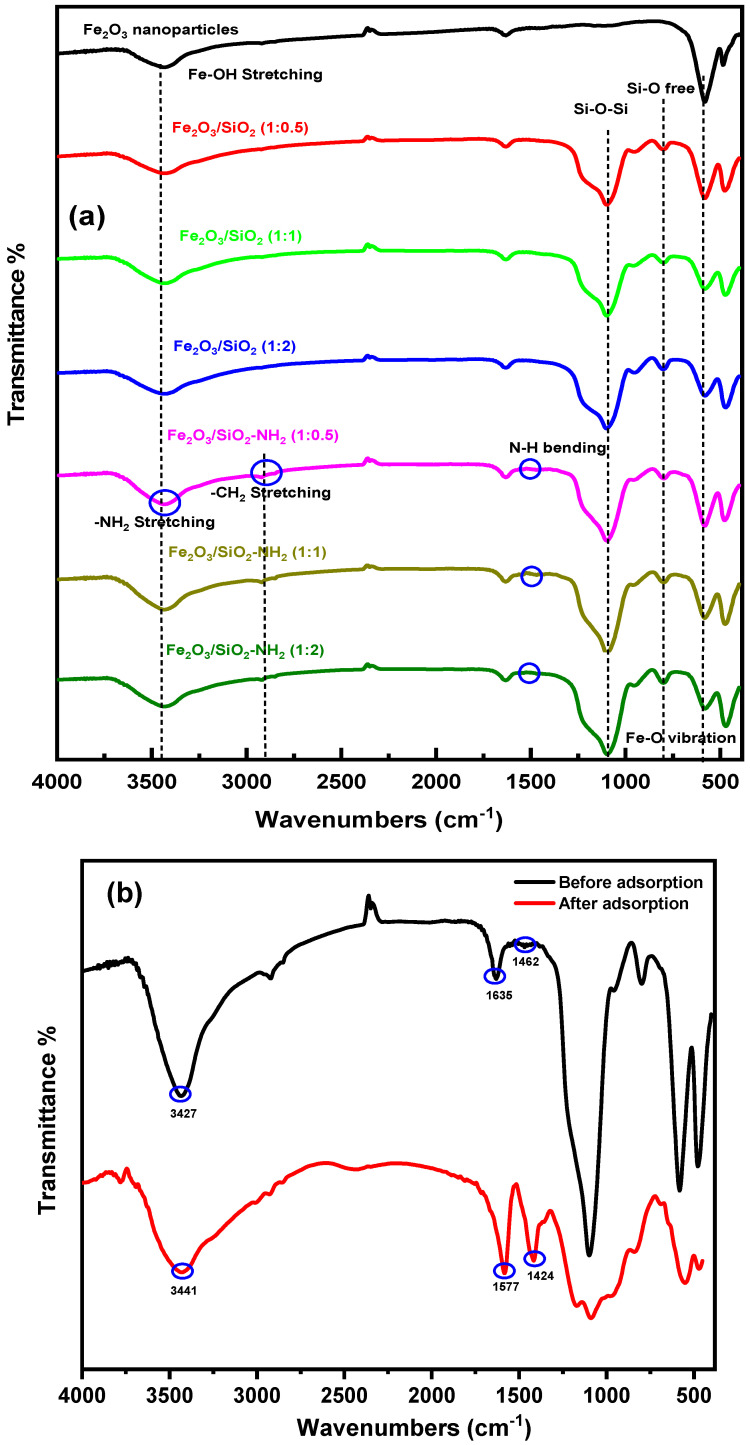
(**a**) FT-IR spectra of Fe_2_O_3_ nanoparticles, Fe_2_O_3_/SiO_2_ developed by incorporating Fe_2_O_3_ with different ratios of TEOS 1:0.5, 1:1 and 1:2 and Fe_2_O_3_/SiO_2_–NH_2_ nanocomposites (**b**) FT-IR spectra of Fe_2_O_3_/SiO_2_–NH_2_ (1:2 ratio) nanocomposite before and after metal ion adsorption.

The FT-IR spectra of Fe_2_O_3_, Fe_2_O_3_/SiO_2_ MNPs, and Fe_2_O_3_/SiO_2_–NH_2_ nanocomposites, which were produced using varying ratios of tetraethyl orthosilicate (TEOS), were measured within the 400–4000 cm^−1^ range and are shown in Figure 2. The Fe–O-stretching vibration band is represented by a significant peak seen at 578 cm^−1^. It is also possible that the stretching vibrations of the Fe–OH groups on the surface are responsible for the strong peak at 3434 cm^−1^. Symmetric and asymmetric bending vibration of the PVP stabilizing agent’s C=O results in an absorption peak at 1645 cm^−1^, indicating the presence of a PVP layer on the surface of Fe_2_O_3_ nanoparticles [38]. The Fe_2_O_3_/SiO_2_ (1:0.5, 1:1, and 1:2 ratio) spectra show three new distinct peaks at range 3445 cm^−1^, 1090 cm^−1^, and 790 cm^−1^, in addition to the bands previously reported in the spectrum of Fe_2_O_3_ nanoparticles. The stretching vibration of the silanol group (Si–OH) coming from hydrogen bonding with the surface’s physisorbed water molecules, the siloxane bond (Si–O–Si), and the free silanol group are represented by these peaks. These findings indicate the presence and effective coating of Fe_2_O_3_ by a silica shell with varying ratios. Furthermore, the peaks observed for Fe_2_O_3_/SiO_2_ exhibited modest variations as a result of the varying quantities of tetraethyl orthosilicate (TEOS) applied to the Fe_2_O_3_ nanoparticles. The presence of the siloxane bond (Si–O–Si) in the Fe_2_O_3_/SiO_2_ spectra became stronger as the proportion of TEOS component (Fe_2_O_3_ and TEOS ratios 1:0.5, 1:1, and 1:2) increased. The rise in surface to volume ratio led to an increase in particle size, which in turn had an effect on the Fe–O-stretching vibration band [39]. Similar distinguishing bands are seen in the Fe_2_O_3_/SiO_2_–NH_2_ spectra as well. Two further weak peaks, at 2860 and 2913 cm^−1^, are still visible, and they are related to the symmetric and asymmetric stretching modes of the –CH_2_ groups in the propyl groups. The bands at 3427 and 1462 cm^−1^, respectively, are responsible for the occurrence of –NH_2_ stretching and –NH bending [40]. The terminal amino group of the APTS molecule exhibits a modest dipole moment, rendering it unobservable. In addition, the loading of APTS onto the silica surface results in the formation of a monolayer, which gives rise to a distinct and concealed distinctive band [41]. The results provided confirmation of the amine functionalization of the hematite/silica composite.

The FT-IR spectra of Fe_2_O_3_/SiO_2_–NH_2_ (1:2 ratio) were measured before and after the adsorption of Hg^2+^ ions to provide additional information on the interaction between Fe_2_O_3_/SiO_2_–NH_2_ (1:2 ratio) and Hg^2+^ ions. The Fe_2_O_3_/SiO_2_–NH_2_ (1:2 ratio) spectra are shown to alter with the adsorption of Hg^2+^ ions, as seen in Figure 2b. A band at 1635 cm^−1^ is displaced to 1577 cm^−1^, and the distinctive peak at 3427 cm^−1^, which corresponds to the -OH and -NH_2_ stretching, is shifted to 3441 cm^−1^ showing the presence and the interaction between adsorbed Hg^2+^ ions and function groups. Fe_2_O_3_/SiO_2_–NH_2_ (1:2 ratio) and Hg^2+^ ions exhibit a significant interaction, as seen by the FT-IR spectra. The interaction between Hg^2+^ions and -NH_2_ groups, which changes the vibrational properties of the -NH bond, may be the cause of the stronger peak corresponding to -NH bending in Fe_2_O_3_/SiO_2_–NH_2_ (1:2 ratio) after adsorption as compared to Fe_2_O_3_/SiO_2_–NH_2_ (1:2 ratio) before adsorption. The spectra indicated changes in the position of bands before and after the Hg^2+^ ions adsorption. The change means that the functional groups of the nanocomposite shifted and remained unchanged on the surface after interacting with the metal ions.

#### 3.1.2. XRD Study

**Figure 3:**
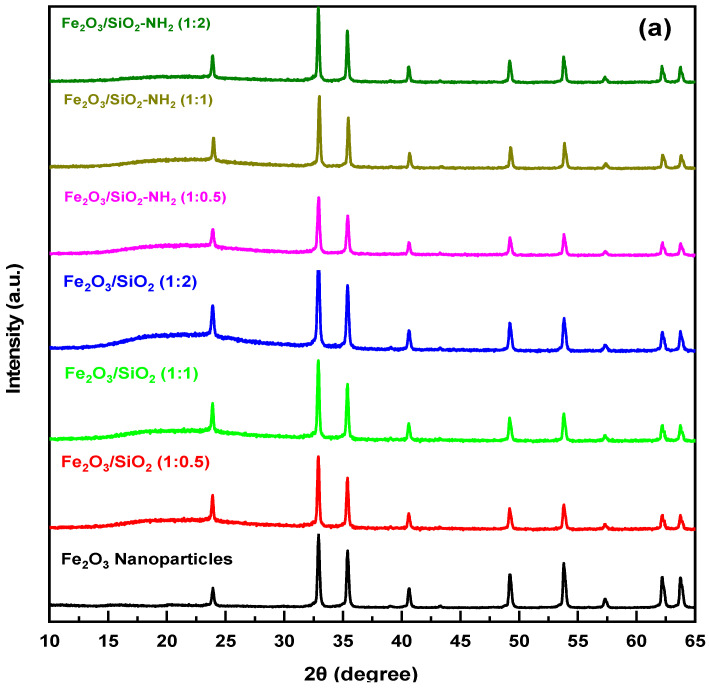

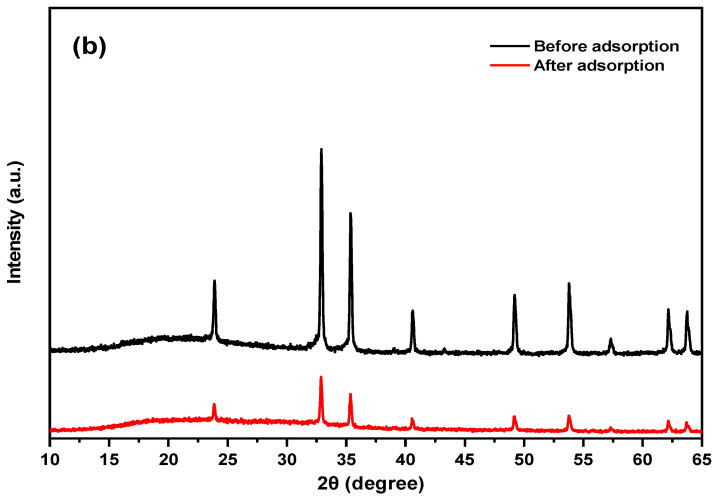
(**a**) X-ray pattern of Fe_2_O_3_ nanoparticles, Fe_2_O_3_/SiO_2_ developed by incorporating Fe_2_O_3_ with different ratios of TEOS 1:0.5, 1:1, and 1:2, respectively, and Fe_2_O_3_/SiO_2_–NH_2_ samples. (**b**) X-ray pattern of Fe_2_O_3_/SiO_2_–NH_2_ (1:2 ratio) nanocomposite before and after metal ions adsorption.

Figure 3 illustrates the XRD analysis of the crystallinity of the Fe_2_O_3_, Fe_2_O_3_/SiO_2_ MNPs, and Fe_2_O_3_/SiO_2_–NH_2_ nanocomposites that were synthesized using varying ratios of TEOS. The Fe_2_O_3_ nanoparticles’ XRD pattern has peaks at 24°, 33°, and 64°, which are related to the (012), (104), and (220) planes of α-Fe_2_O_3_ [38,42]. The γ-Fe_2_O_3_ (311), (400), (422), (511), and (440) peaks at 35, 41, 49, 54, and 62 degrees can be linked to these particles, confirming that the particles are a combination of γ- and α-Fe_2_O_3_ [43,44]. Hematite’s peaks stayed the same following coating with SiO_2_ at varying ratios (1:0.5, 1:1, and 1:2); nevertheless, a broad peak appeared in the 18° to 28° range. This demonstrates that Fe_2_O_3_ is effectively coated with an amorphous SiO_2_ layer in various ratios. The variation in TEOS amounts on the Fe_2_O_3_ nanoparticles caused a little variation in the peaks in the range of 18° to 28° for Fe_2_O_3_/SiO_2_ [45,46]. Furthermore, comparable characteristic peaks were also detected for the Fe_2_O_3_/SiO_2_ and Fe_2_O_3_/SiO_2_–NH_2_ surfaces, as shown in Figure 3a. This indicates that the crystalline phase of the Fe_2_O_3_ nanoparticles remains stable throughout the process of silica coating and functionalization, with no alterations to the topological structure or inherent properties [47]. In addition, an XRD analysis was performed to confirm the material’s crystal integrity before and after the mercury ions were adsorbed. As demonstrated in Figure 3b, the primary peaks before and after adsorption exhibited similar intensities, but there were shifts in the positions of the peaks at 24° and 33°, which corresponded to the (012) and (104) planes of α-Fe_2_O_3_ nanoparticles, respectively. Our study findings indicate that the nanocomposite’s surface experienced chemical adsorption of the Hg^2+^ ions. Furthermore, the material’s crystal structure stays mostly unaltered following the adsorption of mercury, indicating its excellent stability.

#### 3.1.3. SEM and EDS Study

**Figure 4:**
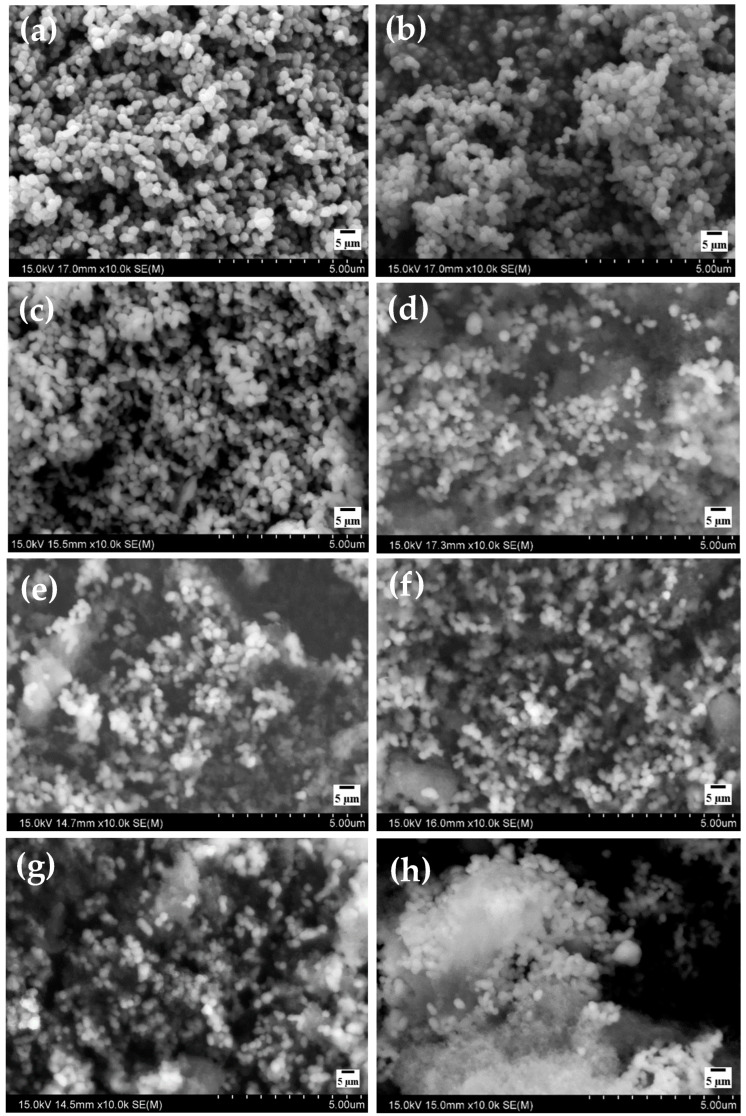
SEM images of (**a**) Fe_2_O_3_ nanoparticles; (**b**–**d**) Fe_2_O_3_/SiO_2_ developed by incorporating Fe_2_O_3_ with different ratios of TEOS 1:0.5, 1:1, and 1:2, respectively; (**e**–**g**) Fe_2_O_3_/SiO_2_–NH_2_ samples; and (**h**) Fe_2_O_3_/SiO_2_–NH_2_ (1:2 ratio) nanocomposite after metal ion adsorption.

Figure 4a demonstrated that the prepared Fe_2_O_3_ nanoparticles have a uniform spherical shape, with an average diameter of about 310 nm. The image also showed that these spherical NPs of Fe_2_O_3_ have a smooth surface with large aggregation. Fe_2_O_3_/SiO_2_ developed by incorporating Fe_2_O_3_ with different ratios of TEOS 1:0.5, 1:1, and 1:2 indicates that the SiO_2_ layer has successfully coated the surface of the Fe_2_O_3_, and by increasing the amount of TEOS, the layer formation from SiO_2_ increased, as can be shown in Figure 4b, c, and d, respectively. After functionalization of prepared materials in Figure 4b–d by APTES, the materials kept their spherical morphology with aggregation less than that which occurred in Figure 4b–d. These results indicated that the functionalization process was achieved successfully and obtained Fe_2_O_3_/SiO_2_–NH_2_ samples, as shown in Figure 4e–g. In Figure 4h, after metal ions adsorption, the Fe_2_O_3_/SiO_2_–NH_2_ (1:2 ratio) nanocomposite shows great change in morphology of the material before adsorption that indicates the adsorption of Hg^2+^ on the surface of Fe_2_O_3_/SiO_2_–NH_2_ (1:2 ratio) nanocomposite.

EDS is the technique used for the elemental analysis of the prepared samples. Table 1 shows the mass percentage of each element in the prepared materials. The data confirm the presence of Fe and O in the Fe_2_O_3_ nanoparticles, while Fe, O, and Si were detected with different percentages in the Fe_2_O_3_/SiO_2_, whose Si percentage increased by increasing the ratio between Fe_2_O_3_ and SiO_2_. The Fe, O, Si, and N in Fe_2_O_3_/SiO_2_–NH_2_ indicate the successful functionalization of Fe_2_O_3_/SiO_2_ with different ratios between Fe_2_O_3_/SiO_2_ and APTMS precursor molecules. Also, by increasing this ratio, the percentage of the N element increased. The amount of amino groups on the surface of Fe_2_O_3_/SiO_2_–NH_2_ (1:0.5 ratio) may be very small, so the absence of a nitrogen (N) peak in Fe_2_O_3_/SiO_2_–NH_2_ (1:0.5 ratio) is anticipated, due to its low Z-number and its overlap with the K-alpha peaks of carbon (C) and oxygen (O) [48]. Finally, the appearance of the percentage of Hg element indicates the occurrence of the adsorption process of Hg element on the surface of Fe_2_O_3_/SiO_2_–NH_2_ (1:2 ratio).

#### 3.1.4. DLS and Zeta Potential Measurement

**Figure 5:**
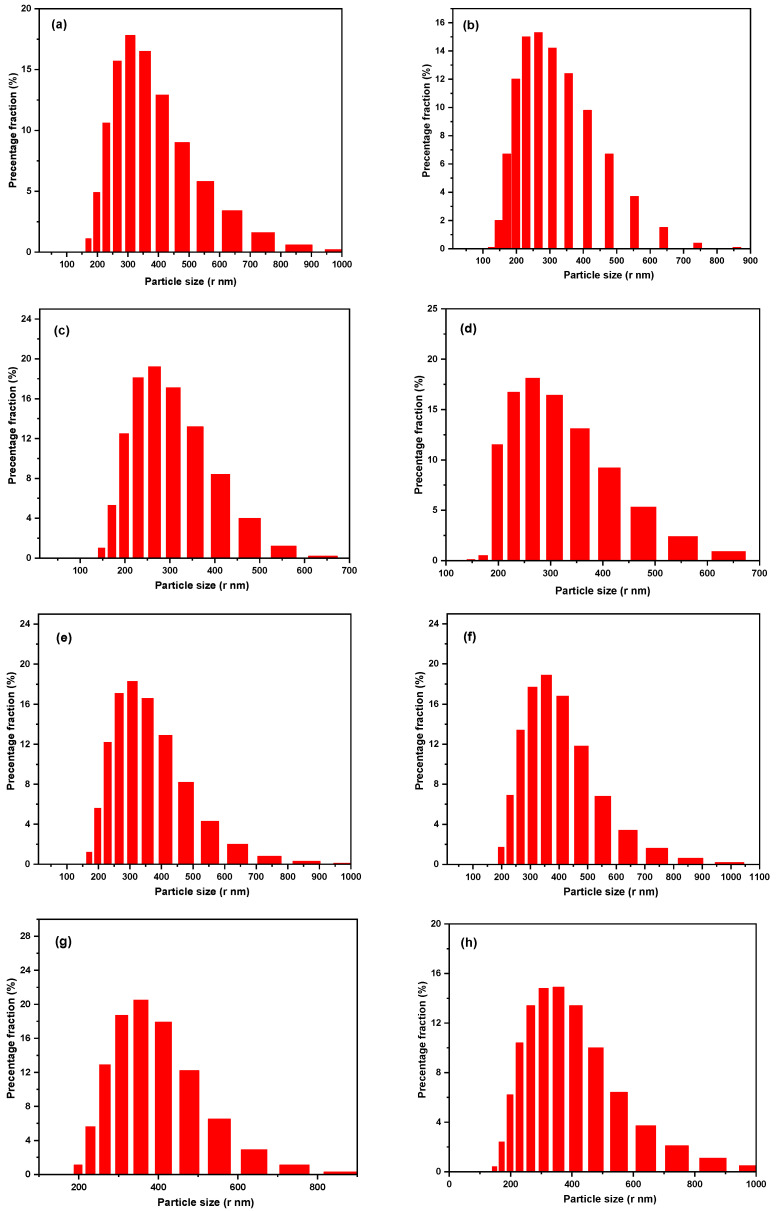
Particle size distribution of (**a**) Fe_2_O_3_ nanoparticles; (**b**–**d**) Fe_2_O_3_/SiO_2_ developed by incorporating Fe_2_O_3_ with different ratios of TEOS, 1:0.5, 1:1 and 1:2, respectively; (**e**–**g**) Fe_2_O_3_/SiO_2_–NH_2_ samples; and (**h**) Fe_2_O_3_/SiO_2_–NH_2_ (1:2 ratio) nanocomposite after metal ions adsorption.

The particle size distributions of the prepared materials were analyzed using the DLS method and are shown in Figure 5. Figure 5a shows the DLS analysis data of the Fe_2_O_3_ nanoparticles that have an average particle size of about 317 nm. The average particle size increased to 373 nm, 383 nm, and 420 nm after incorporating Fe_2_O_3_ with different ratios of TEOS, 1:0.5, 1:1, and 1:2, respectively, indicating that the SiO_2_ shell increases around the Fe_2_O_3_ core with the increasing amount of TEOS, shown in Figure 5b–d. However, these numbers have been observed to increase to average sizes of 462 nm, 485 nm, and 498 nm after being functionalized with APTES through the addition of NH_2_ groups to the previous ratios and obtained Fe_2_O_3_/SiO_2_–NH_2_ samples (Figure 5e–g). The adsorption of Hg^2+^ ions leads to a change in the surface charge of the nanocomposite. As a result, they become less stable and aggregate. As a result, we observe a significant increase in particle size after the adsorption of Hg^2+^ ions on the surface of the Fe_2_O_3_/SiO_2_–NH_2_ sample with ratio (1:2) (Figure 5h). It was discovered that the hydrodynamic diameter determined by the DLS method is greater than the diameter determined by the SEM. The reason for this observation might be due to the inability of DLS to differentiate between constituent and agglomerate particles [49].

An instrument called a zeta potential analyzer is used to find the surface charge of nanoparticles. This measurement is made at pH = 5. Table 2 shows that the Fe_2_O_3_ nanoparticles have a −0.85 mV zeta potential. As a result of the surface of Fe_2_O_3_ being modified by different ratios of TEOS, 1:0.5, 1:1, and 1:2, to obtain different ratios of Fe_2_O_3_/SiO_2_, the zeta potential is decreased to −1.05 mV, −1.36 mV, and −1.45 mV, respectively, and become more negative. This decrease is due to increasing OH groups on the surface of the samples, which increase by increasing the ratio of the addition of TEOS. After performing the functionalization of the previous samples using APTES, the zeta potential still decreases, becomes more negative for all samples, and reached −1.89 mV, −3.34 mV, and −3.50 mV, respectively, in the case of the Fe_2_O_3_/SiO_2_–NH_2_ samples due to NH_2_ groups on the surface of the synthesized samples. Table 2 shows that the Fe_2_O_3_/SiO_2_–NH_2_ (1:2) ratio’s surface charge increases to a more positive value of 1.10 mV. The rationale is that the amino groups on the surface of the particles bound to the Hg^2+^ ions consume the surface’s negative charge during the adsorption process, raising the surface potential. The cause for the relief of agglomeration is tentatively determined to be the interaction between the heavy metal ions and adsorbent groups [50]. Additionally, this demonstrates that Hg^2+^ ions are indeed adsorbed by nano-adsorbents. 

#### 3.1.5. BET Measurement

The BET technique was used to calculate the specific surface area of the produced nanoparticles. This technique depends on detecting the amount of gas that has been adsorbed at a known pressure after the gas has been adsorbed on the particle surface. A known particles count can be used to determine the specific surface area. It is particularly essential for reactions on surfaces, heterogeneous catalysis, and adsorption [51]. The different synthesized particles’ specific surface areas were estimated using the BET technique and collected in Table 3. The synthetic material’s BET surface areas, which are 4.3, 49.9, 68.6, and 72.4 m^2^ g^−1^ for Fe_2_O_3_ nanoparticles, Fe_2_O_3_/SiO_2_ (1:0.5, 1:1, and 1:2 ratio), respectively. The sudden jump between Fe_2_O_3_ and Fe_2_O_3_/SiO_2_ is related to the fact that SiO_2_ may have a porous structure. Additionally, the surface areas of the amino-functionalized nanomaterials are 67.9, 85.7, and 100.1 m^2^ g^−1^ for Fe_2_O_3_/SiO_2_–NH_2_ (1:0.5 ratio), Fe_2_O_3_/SiO_2_–NH2 (1:1 ratio), and Fe_2_O_3_/SiO_2_–NH_2_ (1:2 ratio), respectively. Pollutant adsorption is generally favorably facilitated by materials with larger specific surface areas which could increase the capacity for adsorption, so we used Fe_2_O_3_/SiO_2_–NH_2_ (1:2 ratio) to remove mercury ion from the aqueous solution.

#### 3.1.6. VSM Study

**Figure 6:**
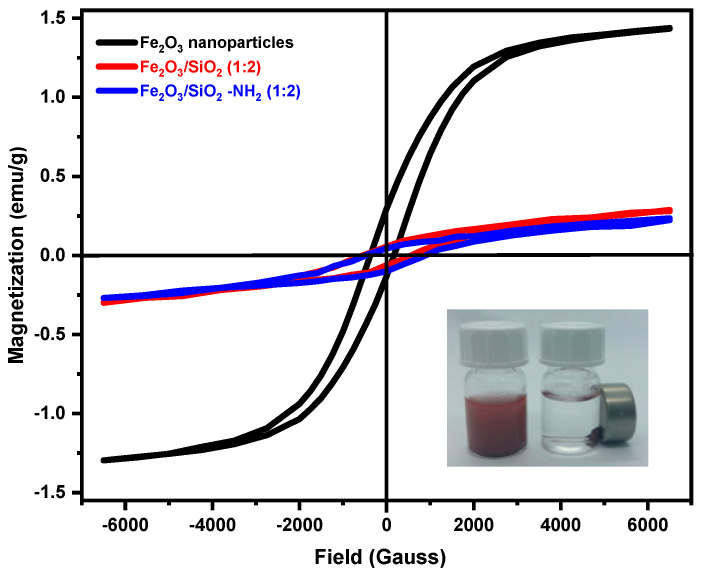
Vibrating sample magnetometer (VSM) for Fe_2_O_3_ NPs, Fe_2_O_3_/SiO_2_ (1:2 ratio), and Fe_2_O_3_/SiO_2_–NH_2_ (1:2 ratio) nanocomposite.

The VSM technique was used to evaluate the magnetic characteristics of Fe_2_O_3_, Fe_2_O_3_/SiO_2_ (1:2 ratio), and their amino-functionalized Fe_2_O_3_/SiO_2_–NH_2_ (1:2 ratio) at room temperature under an external magnetic field varying between −7 kOe and +7 kOe. The M–H hysteresis loop for the samples and the associated saturation magnetization values are shown in Figure 6. Table 4 lists the values of Ms (emu g^−1^), coercivity Hc (Oe), and remanent magnetization Mr (emu g^−1^) that are obtained by analyzing the individual M–H loops. The hysteresis loop reveals that Fe_2_O_3_ nanoparticles have paramagnetic behavior with a substantial saturation magnetic moment (M_s_) value of 1.22 emu∙g^−1^ when subjected to a magnetic field of 20 kG. It is accompanied by a low magnetic remanence (M_r_ = 0.21 emu g^−1^) and low coercivity (H_c_); this indicates that the hematite nanoparticles have α and γ Fe_2_O_3_ types [52,53,54]. When Fe_2_O_3_ is coated with silica with a 1:2 ratio as Fe_2_O_3_/SiO_2_ nanocomposite, there is a decrease in both M_s_ (magnetization saturation) and M_r_ (remanence magnetization) and an increase in and H_c_ (coercivity), as can be seen in Table 4. Additionally, there is a little decrease in the M_s_ values in the samples after amino functionalization, which can be attributed to the limited impact of silica and amino groups on the total mass of magnetic nanoparticles. Consequently, the presence of coated Fe_2_O_3_ in nanocomposites ensures that the adsorbents remaining have magnetic properties, facilitating their effortless separation from the aqueous solution using an external magnet; a reduction in the M_s_ value may indicate a lower proportion of net magnetic material per gram of the entire sample [55].

#### 3.1.7. TGA Study

**Figure 7:**
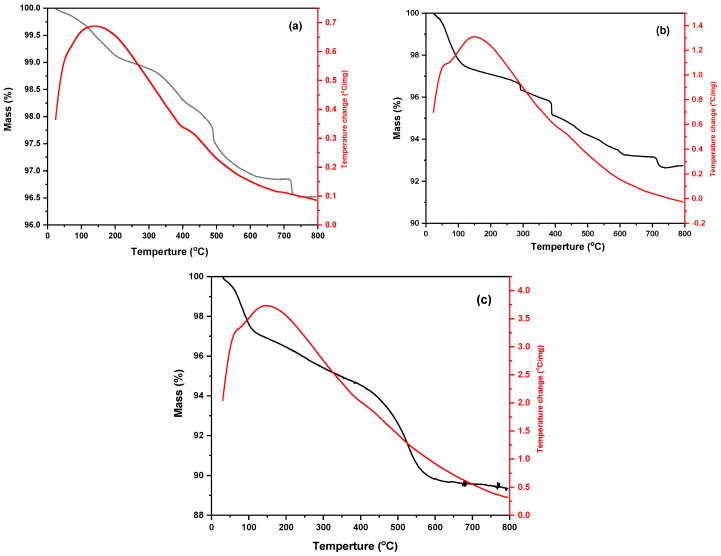
DSC–TGA of (**a**) Fe_2_O_3_ nanoparticles, (**b**) Fe_2_O_3_/SiO_2_ developed by incorporating Fe_2_O_3_ with a ratio of 1:2, (**c**) Fe_2_O_3_/SiO_2_–NH_2_ with a ratio of 1:2.

A thermal analysis technique called thermogravimetric analysis (TGA) examines changes in a material’s chemical and physical properties in relation to a steady temperature increase or a constant temperature and mass loss over time. Data on physical events, including vaporization, sublimation, adsorption, desorption, and absorption, are provided by TGA [53]. The evaluation of the thermal properties of the synthesized samples was the primary purpose of the thermogravimetric analysis (TGA) and differential scanning calorimetry (DSC) analysis [56]. Figure 7 shows the DSC–TGA of (a) Fe_2_O_3_ nanoparticles, (b) Fe_2_O_3_/SiO_2_ developed by incorporating Fe_2_O_3_ with ratio 1:2, (c) Fe_2_O_3_/SiO_2_–NH_2_ with a 1:2 ratio. The synthesized materials show an exothermic peak from the DSC pattern at around 140 and 410 °C for Fe_2_O_3_ nanoparticles, 58 °C and 148 °C for Fe_2_O_3_/SiO_2_ developed by incorporating Fe_2_O_3_ with a 1:2 ratio, and 65 °C and 150 °C for Fe_2_O_3_/SiO_2_–NH_2_ with a 1:2 ratio. As it can be observed from the TGA curves (Figure 7), the weight losses of (a) Fe_2_O_3_ nanoparticles, (b) Fe_2_O_3_/SiO_2_ developed by incorporating Fe_2_O_3_ with the 1:2 ratio, (c) Fe_2_O_3_/SiO_2_–NH_2_ with the 1:2 ratio, as the prepared materials had been heated up to 800 °C, have been 4%, 8%, and 11%, respectively.

In detail, three different mass loss phases are shown for the Fe_2_O_3_ nanoparticles in Figure 7a over temperature ranges. At 214 °C, the initial phase of weight loss took place. The elimination of water that exists at the surface of Fe_2_O_3_ is responsible for the 1% mass loss. The combustible organic compounds in the sample cause a mass loss of 1.40%, which occurs at 490 °C in the second stage [57]. At 717 °C, there is a weight loss of 0.84% in the third stage, which could be attributed to the synthesized compounds’ transition phase caused by the decomposition of Fe_2_O_3_ into Fe_3_O_4_ [57,58]. Figure 7b for Fe_2_O_3_/SiO_2_ developed by incorporating Fe_2_O_3_ with 1:2 ratio, the weight loss that happened at 114 °C was 2.44%, and the elimination of water that was previously present on the surface is responsible for this weight loss. Because of the molecules of ethanol and water that have been physically adsorbed and attached to the surface, the main weight loss only happens below 400 °C. Additionally, at a temperature of ˃400 °C, an about 2.4% weight loss was noted, which was related to the hydrophilicity and degradation of physisorption molecules on the surface of silicon oxide [59]. Figure 7c shows Fe_2_O_3_/SiO_2_–NH_2_ with a ratio of 1:2 heating to 115 °C; the observed mass loss is consistent with absorbed moisture evaporating and residue from NH_4_OH. Weight loss occurs when aminopropyl (NH_2_(CH_2_)_3_-) groups are removed from the surfaces of the nanoparticles and the residual siloxane groups (Si–O–Si) break as a result of heating to 428 °C and 600 °C [59,60].

### 3.2. Adsorption Study

#### 3.2.1. Effects of the Adsorbent Type on the Adsorption Efficiency

**Figure 8:**
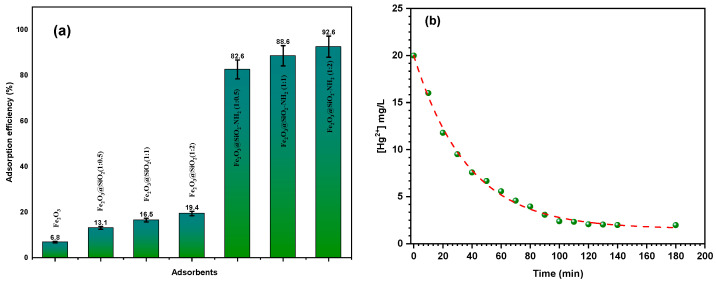
(**a**) The adsorption efficiency of Hg^2+^ ions by Fe_2_O_3_, Fe_2_O_3_/SiO_2_, and their Fe_2_O_3_/SiO_2_–NH_2_ as adsorbents. (**b**) The concentration changes of Hg^2+^ ions in solution during adsorption on Fe_2_O_3_/SiO_2_–NH_2_ (1:2 ratio).

This adsorption research aims to identify the best available adsorbent for removing Hg^2+^ ions from an aqueous solution. The study compares the activity of Fe_2_O_3_ and Fe_2_O_3_/SiO_2_, which were synthesized using varying ratios of TEOS (1:0.5, 1:1, and 1:2), as well as their amino-functionalized counterparts. The removal efficiencies of these adsorbents were examined using identical conditions, including an initial mercury concentration ([Hg^2+^]_o_) of 20 mg L^−1^, a dosage of 10 mg, a pH of 5.0 ± 0.1, a temperature of 30 °C, and a stirring speed of 140 rpm. The results depicted in Figure 8a demonstrate the removal efficiency of Fe_2_O_3_, Fe_2_O_3_/SiO_2_, and their Fe_2_O_3_/SiO_2_–NH_2_ adsorbents towards Hg^2+^ ions. The removal efficiencies for Hg^2+^ ions were found to be 6.8%, 13.1%, 16.5%, 19.4%, 82.6%, 88.6%, and 92.6% for Fe_2_O_3_; Fe_2_O_3_/SiO_2_ with TEOS ratios of 1:0.5, 1:1, and 1:2; and their Fe_2_O_3_/SiO_2_–NH_2_ adsorbents, respectively. Based on these findings, it is evident that Fe_2_O_3_/SiO_2_–NH_2_ with a ratio of 1:2 is a superior adsorbent compared to other surfaces in the process of removing metal ions. The observed results can be attributed to the augmentation in surface area following the process of encapsulation by a silica layer, which increased from 4.26 to 72.4 m^2^/g. Subsequently, the surface area further increased to 100.1m^2^/g after the amino functionalization, which resulted in a smoother surface, leading to an increase in the amount of adsorption-active sites (specifically, grafted amino saline groups). Therefore, the Fe_2_O_3_/SiO_2_–NH_2_ with a ratio of 1:2 was selected as the primary adsorbent in this investigation for the purpose of eliminating metal ions, as shown in Figure 8b.

#### 3.2.2. Effect of Contact Time on the Adsorption Efficiency

The impact of the contact time on the Fe_2_O_3_/SiO_2_–NH_2_ (1:2 ratio) removal of Hg^2+^ ions from a solution containing 20 mg L^−1^ of Hg^2+^ ions using 10 mg of Fe_2_O_3_/SiO_2_–NH_2_ (1:2 ratio) adsorbent at pH 5.0 ± 0.1 and 30 °C is depicted in Figure 8b. The figure illustrates how quickly ions are removed during the first stage, which lasts from 0 to 20 min. The procedure achieves a saturated state after the second step, which can be completed at a slower pace for 40 to 90 min. This could be because, during the first stage, there are many more available unoccupied surface sites in the Fe_2_O_3_/SiO_2_–NH_2_ (1:2 ratio), which causes the Hg^2+^ ions to be adsorbed on the outer surface relatively quickly [61]. With 92.6% of the Hg^2+^ ions adsorbed, the figure illustrates how the adsorption process reached equilibrium in 180 min.

#### 3.2.3. Effects of Dose on the Adsorption Efficiency

**Figure 9:**
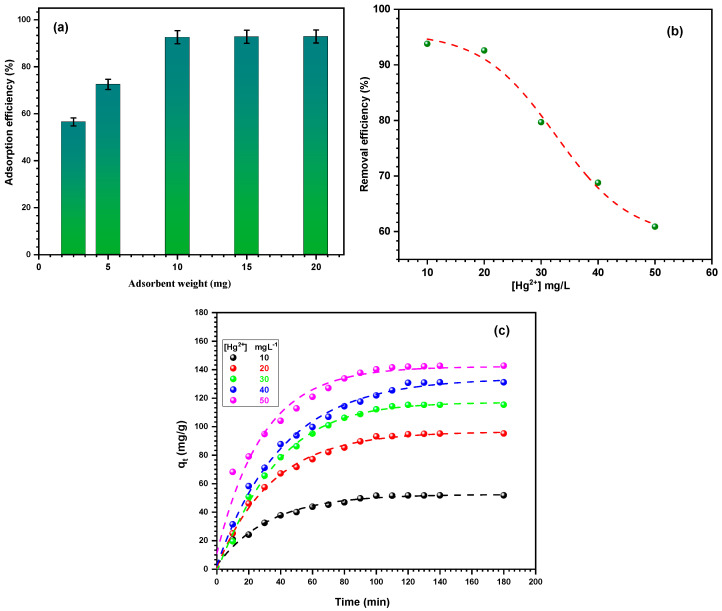
(**a**) The effect of adsorbent dosage on the adsorption efficiency of Hg^2+^ ions, (**b**) effect of initial concentration on the adsorption efficiency of Hg^2+^ ions, and (**c**) effect of initial concentration on the adsorption capacity of Hg^2+^ ions onto Fe_2_O_3_/SiO_2_–NH_2_ with a ratio of 1:2.

The amount of adsorbent present in the solution is a crucial component that influences the rate of adsorption. The impact of Fe_2_O_3_/SiO_2_–NH_2_ dosage with a ratio of 1:2 was assessed within the range of 2.5 to 25 mg. The concentration of mercury ions was kept constant at 20 mg L^−1^, with a pH of 5.0 ± 0.1 and a temperature of 30 °C. The stirring speed was maintained at a constant rate of 140 rpm. The findings show that Fe_2_O_3_/SiO_2_–NH_2_ is capable of effectively eliminating Hg^2+^ ions at a minimal dose, indicating that it has sufficient active sites for adsorbing Hg^2+^ ions, even when they are present in low concentrations. In addition, the findings indicated that when the nanocomposite weight increased from 2.5 to 10 mg, the removal efficiency of Hg^2+^ ions increased from 56.5 to 92.6%, as shown in Figure 9a. This phenomenon may be explained by the expanding surface area of the nanocomposite, which leads to a greater number of active sites for metal ions to be adsorbed. The removal efficiency of Hg^2+^ ions increase fast as the dosage increases until all the ions are adsorbed on the nanocomposite surface. At this point, the clearance efficiency stabilizes at a value of 10 mg, suggesting that no more adsorptions occur [62]. Hence, the 10 mg dosage was chosen as the standard dose for this investigation.

#### 3.2.4. Effect of Initial Metal Ion Concentration on the Adsorption Efficiency

The impact of the initial Hg^2+^ ion concentration was examined at a fixed dosage of 10 mg of Fe_2_O_3_/SiO_2_–NH_2_ with a ratio of 1:2, and at different Hg^2+^ ion concentrations ranging from 10 to 50 mg L^−1^, and the results are shown in Figure 9b,c. This outcome demonstrated that in response to a rise in the initial concentration of Hg^2+^ ions, sorption efficiency and adsorption capacity exhibited opposing tendencies. The removal efficiency of Hg^2+^ ions decreased from 93.80 to 59.29% when the concentration of Hg^2+^ ions was raised from 10 to 50 mg L^−1^, as shown in Figure 9b. Hg^2+^ ions adsorb quickly on the Fe_2_O_3_/SiO_2_–NH_2_ surface in the early stages, and at the greatest concentration of Hg^2+^ ions (50 mg L^−1^), this rate is dramatically decreased, as shown in Figure 9b. In contrast to the small number of ions at low concentrations, this characteristic is associated with the greatest accessible abundance of adsorption sites on the adsorbent surface. On the other hand, a significant drop in the removal efficiency occurs at high concentrations when an additional population of ions encounters a finite number of adsorption sites. The previously adsorbed ions’ blockage of active sites against the remaining ions in the solution is the cause of this decline. The adsorption of metal ions can be influenced by various parameters, such as surface charge, adsorbent surface properties, hydrophilic and hydrophobic features, van der Waals forces, electrostatic interaction, hydrogen bonding, and other factors [63]. Anticipatingly, an increase in the Hg^2+^ concentration causes a limited adsorption capacity to prevent additional metal ion adsorption, resulting in a drop in the overall removal percentage.

#### 3.2.5. Effect of pH and Zeta Potential on the Adsorption Efficiency

In adsorption studies, pH is a crucial variable that can have a big impact on the outcomes. The pH environment may impact the morphology and charge state of mercury ions in the solution, which can impact how the ions interact with the adsorbent surface and the overall adsorption effect. Consequently, batch adsorption studies are required to examine the adsorption capacity of the adsorbent under various pH environments and to analyze the association between the pH value and the adsorption performance of the adsorbent.

**Figure 10:**
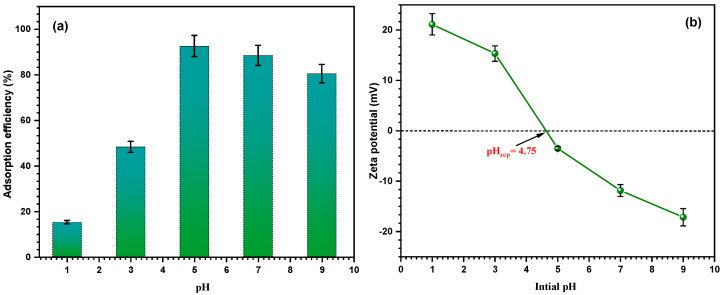
(**a**) The effect of pH on the adsorption efficiency and (**b**) the zeta potential of Fe_2_O_3_/SiO_2_–NH_2_ at different pH solutions.

The zeta potential and mercury removal efficiency of Fe_2_O_3_/SiO_2_–NH_2_ under a pH gradient range (1–9) were demonstrated in Figure 10a,b; the concentration of mercury ions was kept constant, at 20 mg L^−1^, with a dosage of 10 mg and a temperature of 30 °C. When the pH rose from 1.0 to 9.0, the adsorbent often displayed a pattern where the removal first increased to 5 and then dropped. This was due to the fact that at a lower pH, the solution’s hydrogen and Hg^2+^ ions were in competition for binding with the adsorption sites on the surface of the adsorbent, producing inadequate removal rates. Furthermore, it was easy to observe that the adsorbent’s zero-charge point was 4.75, indicating that when pH < 4.75, the adsorbent’s positively charged surface produced electrostatic repulsion with the Hg^2+^ ions in the solution [64]. The concentration of hydrogen ions steadily drops as the pH rises, making the mercury ions’ competitive advantage clear. Moreover, the adsorbent obtained its greatest removal rate at pH = 5 due to the soft-base nature of its nitrogenous functional groups, which made it more friendly with softer mercury ions [65]. Furthermore, at pH = 5, the negatively charged adsorbent surface produced electrostatic adsorption for the Hg^2+^ ions present in the solution. The adsorption effectiveness drops, most likely as a result of the chemical precipitation of metal ions, when the pH rises higher because the mercury ions in the solution form hydroxides and are harder for the adsorbent to capture. Consequently, the adsorption studies are carried out at the ideal pH of 5, which prevents chemical precipitation. Figure 10b displays the Fe_2_O_3_/SiO_2_–NH_2_ zeta potentials. The degree of protonation of the material’s nitrogen functional groups reduces as the pH rises, changing the material’s zeta potential from positive to negative. Furthermore, Fe_2_O_3_/SiO_2_–NH_2_ exhibits a point of zero charges at a pH of 4.75, indicating that the material is negatively charged in weakly acidic and alkaline environments.

### 3.3. Adsorption Kinetics Studies

The rate of adsorption is an essential factor in evaluating the viability of employing an adsorbent for extensive applications. This experiment assessed the efficacy of the adsorbent by utilizing a time gradient. Three kinetic models, both linearized and non-linearized, were used to study the adsorption process. These models include the pseudo-first-order, pseudo-second-order, and intraparticle diffusion models, which are represented by Equations (4–8) in Table 5. The purpose was to determine the dominant mechanism that governs the adsorption process. The adsorption of Hg^2+^ ions onto Fe_2_O_3_/SiO_2_–NH_2_ (1:2) was investigated over time, using various beginning concentrations within the linear concentration range of Hg^2+^ ions [66,67]. The model’s fitness for the adsorption process was determined based on the highest regression coefficient R^2^ values.

*q_t_* and *q_e_* (mg g^−1^) represent the amount of Hg^2+^ ions adsorbed at a specific contact time *t* and at equilibrium, respectively. The rate constants *k_1_* (min^−1^), *k_2_* (g mg^−1^), and *k_p_* (mol/g min ^0.5^) represent the rates of (PFO), (PSO), and intraparticle diffusion, respectively. The constant value *C* (mg g^−1^) denotes the thickness of the boundary layer.

**Figure 11:**
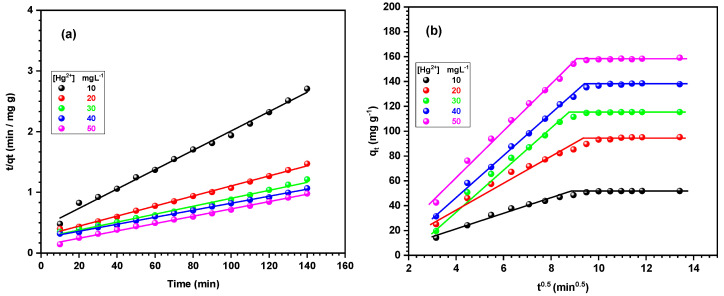
Adsorption kinetics plots of (**a**) pseudo-second-order and (**b**) intraparticle diffusion for adsorption of Hg^2+^ ions on Fe_2_O_3_/SiO_2_–NH_2_ nanocomposite (initial conc. Of Hg^2+^ ions: 20 mg L^−1^, Fe_2_O_3_/SiO_2_–NH_2_: 10 mg, Hg^2+^ ions solution: 50 mL, shaking speed: 140 rpm, temperature: 30 °C, pH = 5.0 ± 0.1).

The kinetic parameters, correlation coefficient R^2^, and calculated adsorption capacity (*q_e_*, cal) were determined based on the data from Figure 11a,b and are presented in Table 6. Table 6 indicates that the adsorption of Hg^2+^ ions onto the adsorbent did not conform to the pseudo-first-order model because of the poor correlation coefficient. Furthermore, the values of (*q_e_*, cal) and (*q_e_*_,_ exp) were incompatible. Nevertheless, the computed value (*q_e_*, cal) obtained from the pseudo-second-order equation exhibited a strong agreement with the experimental value (*q_e_*_,_ exp). Furthermore, the value of (R^2^) was nearly equal to one, suggesting that the pseudo-second-order model is the most suitable to explain the adsorption of Hg^2+^ ions from an aqueous solution onto the surface of Fe_2_O_3_/SiO_2_–NH_2_. The applicability of the pseudo-second-order model in processing suggests that the adsorption mechanism was primarily influenced by chemical adsorption [68].

The fitting data of the Intraparticle diffusion model exhibited two linear trends, as depicted in Figure 11b, suggesting that the adsorption mechanism comprised two distinct steps rather than a single step. The sequential nature of each phase suggests that intraparticle diffusion is occurring. The initial step mostly resulted from film diffusion, with the fitting line deviating from the origin, indicating that the adsorption of Hg^2+^ ions took place on the exterior surface. The second stage involved the allocation of intraparticle diffusion of ions, which subsequently bound to the internal adsorption sites. K_p_ demonstrated the driving power of the Fe_2_O_3_/SiO_2_–NH_2_ (1:2) adsorption process. The results of the fitting, as presented in Table 6, indicate that the first step has the highest K_p_ value, while the second step has the lowest. The rapid adsorption rate of Hg^2+^ ions is attributed to the high concentration of Hg^2+^ ions and the abundance of adsorption sites on the surface of Fe_2_O_3_/SiO_2_–NH_2_. Subsequently, as a result of the exhaustion of the adsorption sites on the Fe_2_O_3_/SiO_2_–NH_2_ surface, Hg^2+^ ions began to spread out towards the adsorption sites located within the pores. This led to a slower adsorption process and a reduced adsorption force. Ultimately, the adsorption process reaches a state of equilibrium. These results indicate that the adsorption mechanism of Fe_2_O_3_/SiO_2_–NH_2_ is governed by particle diffusion, suggesting a chemical reaction [69].

### 3.4. Adsorption Isotherm Models

To understand more about the distribution of adsorbate molecules between the liquid and solid phases at equilibrium, the adsorption isotherm was investigated. The adsorption isotherm experiment is an essential technique used to analyze experimental data, using many isotherm models in order to understand the adsorption phenomenon and accurately measure the adsorption capacity of the composite material. This study focused on examining the adsorption of metal ions onto the surface of Fe_2_O_3_/SiO_2_–NH_2_ (1:2) utilizing commonly used linear and non-linear isotherm models, namely Langmuir, Freundlich, Tempkin, and Dubinin–Radushkevich. These models are represented by Equations (9)–(14) presented in Table 7 [70,71,72].

**Figure 12:**
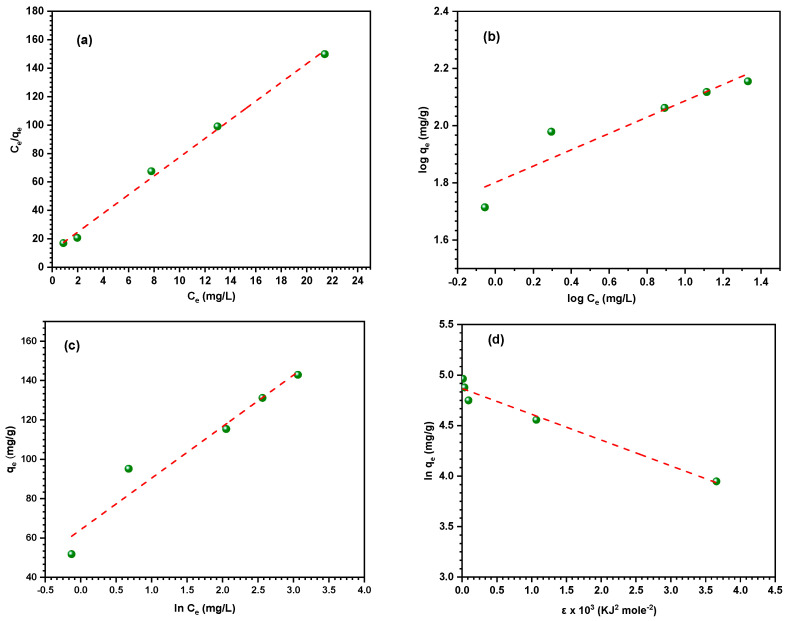
Adsorption isotherm models of Hg^2+^ ions on Fe_2_O_3_/SiO_2_–NH_2_ nanocomposite. (**a**) Langmuir, (**b**) Freundlich, (**c**) Tempkin, and (**d**) Dubinin–Radushkevich (initial conc. Of Hg^2+^ ions: 20 mg L^−1^, Fe_2_O_3_/SiO_2_–NH_2_: 10 mg, Hg^2+^ ions solution: 50 mL, shaking speed: 140 rpm, temperature: 30 °C, pH = 5.0 ± 0.1).

*C_e_* (mg L^−1^) denotes the equilibrium concentration of Hg^2+^ ions in the solution. The variable *q_e_* (mg g^−1^) represents the equilibrium adsorption capacity of Hg^2+^ ions, q_m_ denotes the maximum adsorption capacity, and *K_L_* refers to the Langmuir adsorption constant. The Freundlich constant, *K_F_* (mg g^−1^), represents the level of adsorption intensity, while 1/n indicates the degree of adsorption favorability. The Temkin equilibrium constant, *K_T_* (expressed in L/g), is associated with the highest binding energy. *B* (measured in J mol^−1^) represents the heat of adsorption and is determined using the following expression B = RT/b; *R*. In this equation, T represents the absolute temperature in Kelvin, R represents the gas constant, and b represents the adsorption potential [73]. The theoretical saturation capacity of the Fe_2_O_3_/SiO_2_–NH_2_ nanocomposite is represented by q_s_ (mg g^−1^), while the Polanyi sorption potential is denoted by ε (kJ mol^−1^). The value of ε can be expressed in the following manner: the equation is given by ε = RT ln [1 + 1/Ce]. The slope of the D–R plot (Figure 12d) provides the value of β, which is used to determine the mean adsorption energy (E, kJ mol^−1^) using the following equation:

(15) $$E=\frac{1}{({2\beta )}^{0.5}}$$

Figure 12a–d displays the adsorption isotherms of Langmuir, Freundlich, Tempkin, and Dubinin–Radushkevich (D–R). Table 8 provides the values acquired from the isotherm models, as well as their correlation coefficient (R^2^). The Langmuir model exhibited a stronger correlation (R^2^ = 0.996) with the adsorption data of Hg^2+^ ions, indicating a better fit. On the other hand, the Freundlich and Temkin isotherms showed lower correlation values (R^2^ = 0.844, 0.930), suggesting less agreement with the experimental data. The Langmuir isotherm exhibits a strong correlation with the experimental results. This indicates that the adsorbents possess a surface that is structurally homogeneous, and the primary mechanism involved in adsorption is the formation of a monolayer. Furthermore, the Fe_2_O_3_/SiO_2_–NH_2_ adsorbent exhibited superior adsorption capability, measuring 152.03 mg g^−1^. In addition, the dimensionless factor R_L_ was calculated to assess the feasibility of adsorbing Hg^2+^ ions on the surface of Fe_2_O_3_/SiO_2_–NH_2_. The factor is determined by Equation (16):

(16) $$R_{L}=\frac{1}{1+K_{L}C_{e}}$$

The properties of Hg^2+^ ions adsorption can be determined based on the *R_L_* value. Adsorption is deemed favorable when the value of R_L_ falls within the range of 0 to 1. Adsorption is deemed unfavorable when the value of R_L_ is larger than 1. Adsorption exhibits linearity when the value of *R_L_* is 1. Finally, when the value of R_L_ is 0, the process of adsorption becomes irreversible. The R_L_ value of 0.16 obtained in this work suggests that the adsorption of Hg^2+^ ions on the surface of the Fe_2_O_3_/SiO_2_–NH_2_ magnetic composite is highly favorable.

The Freundlich model revealed that the value of 1/n is 0.285, indicating that Hg^2+^ ions have a preference for adsorption on the surface of the Fe_2_O_3_/SiO_2_–NH_2_ nanocomposite [74]. To differentiate between physical and chemical adsorption, the sorption data were evaluated utilizing the Dubinin–Radushkevich equation. The computed value of E from the D–R model provides insights into the adsorption mechanism, indicating whether it is of a physical or chemical nature. Thus, when the energy (E) is less than 8 kJ mol^−1^, the adsorption is classified as physical. Chemical adsorption is referred to when the value of E falls between 8 and 16 kJ mol^−1^ or higher than this range [75]. The adsorption energy of 9.97 kJ mol^−1^ (as seen in Table 8) confirms the chemical adsorption of Hg^2+^ ions on the surface of the Fe_2_O_3_/SiO_2_–NH_2_ nanocomposite. This result verifies that the adsorption of Hg^2+^ ions from an aqueous solution using Fe_2_O_3_/SiO_2_–NH_2_ occurred through complexation between the amino groups on the surface of the composite and metal ions.

### 3.5. Thermodynamic Parameters

Understanding the effects of temperature on adsorption clarifies the behavior and mechanism of adsorption. The enthalpy change (Δ*H*°), entropy change (Δ*S*°), and free energy of adsorption (Δ*G*°) are examples of thermodynamic parameters that can shed light on the spontaneity and heat change in the adsorption process. The values of both Δ*H*° and Δ*S*° were determined by analyzing the linear relationship between ln (q_e_/C_e_) and 1/T, as described in Equation (17). Meanwhile, the value of Δ*G*° was determined using Equation (18) [76]:

(17) $$\ln\frac{q_{e}}{C_{e}}=\frac{{△S}^{o}}{R}-\frac{{△H}^{o}}{RT}$$

(18) $${△G}^{o}={△H}^{o}-T{△S}^{o}$$

The symbols *q_e_* (mg/g) and *q_t_* (mg/g) are the adsorption capacities of the adsorbent at the equilibrium and definite time, T (K) represents the absolute temperature, and *R* (J/mol K) represents the gas constant. The thermodynamic parameters have been computed and consolidated in Table 9. The adsorption of the Hg^2+^ ions to the Fe_2_O_3_/SiO_2_–NH_2_ (1:2) nanocomposite is characterized by an endothermic process, as indicated by the positive ∆*H*° value. Additionally, it has been shown that the Δ*H*° value provides useful insights about the kind of adsorption. The Δ*H*° values for physical adsorption typically fall within the range of 2.1 to 20.9 kJ mol^−1^, whereas for chemical adsorption, they vary from 20.9 to 418.4 kJ mol^−1^ [77,78]. The Δ*H*° value (41.84 kJ mol^−1^) in this investigation indicates that the synthesized materials could potentially remove Hg^2+^ ions through chemical adsorption. The negative values of Δ*G*° indicate that the Hg^2+^ ions adsorption on the adsorbent occurs spontaneously. On the other hand, the positive values of Δ*S*° suggest that there is an increase in randomness at the interface of the Fe_2_O_3_/SiO_2_–NH_2_ nanocomposite and Hg^2+^ ions throughout the adsorption process. This is the typical result of the chemisorption phenomenon that occurs during the process of adsorption [79].

### 3.6. Selectivity and Reusability of the Nanocomposite

**Figure 13:**
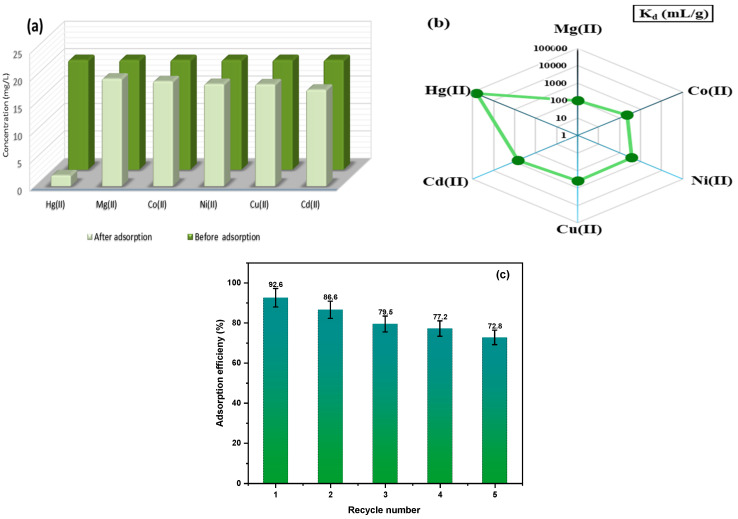
(**a**) Selectivity, (**b**) distribution coefficient (K_d_) of Fe_2_O_3_/SiO_2_–NH_2_ toward different metal ions, and (**c**) recyclability of Fe_2_O_3_/SiO_2_–NH_2_ (1:2) nanocomposite for the removal of Hg^2+^ ions from solution.

To assess the practical use of an adsorbent, it is crucial to examine its capacity to endure challenging conditions that contain various contaminants. To demonstrate the selectivity of the Fe_2_O_3_/SiO_2_–NH_2_ nanocomposite towards different metal ions, selective adsorption studies were conducted. The tests involved using the same concentration of metal ions, such as Mg^2+^, Zn^2+^, Ni^2+^, Cd^2+^, Cu^2+^, and Hg^2+^, in an aqueous solution with a pH of 5.0. The experiments were conducted at a constant temperature of 30 °C for 24 h. The data obtained from Figure 13a demonstrate that the Fe_2_O_3_/SiO_2_–NH_2_ nanocomposite exhibits significant adsorption of Hg^2+^ ions while displaying minimal adsorption of other metal ions. The outcome suggests that Fe_2_O_3_/SiO_2_–NH_2_ demonstrates effective and selective adsorption of Hg^2+^ ions in complex polyionic solutions. In order to explore the impact of the material on different ions, the distribution coefficient (*K_d_*) and selectivity coefficient (*K_s_*) were calculated and compared for each metal ion using Equations (19) and (20), respectively [80,81].

(19) $$K_{d}=\frac{Q}{C_{e}}=\frac{C_{i}-C_{e}}{C_{e}}\cdot \frac{V}{m}$$

(20) $$K_{s}=\frac{K_{d({Hg}^{2+})}}{K_{d(coexisting\;ions)}}$$

where *K_d_* is the distribution coefficient and *K_s_* is selectivity coefficient.

The resultant findings are contained in Table 10. Figure 13b unequivocally illustrated that the distribution coefficient of Hg^2+^ ions (*K_d_* = 62,560 mL/g) was larger than another interfering ion, implying that the adsorbent exhibits a pronounced affinity for mercury ions compared to other ions, following the sequence Hg^2+^ > Cu^2+^ > Cd^2+^ > Ni^2+^ > Zn^2+^ > Mg^2+^ ions. Moreover, the selectivity coefficient of ions suggests that they have minimal impact on the trapping of mercury ions during the adsorption process. Fe_2_O_3_/SiO_2_–NH_2_ nanocomposite exhibits superior selectivity for sorbing Hg^2+^ ions while demonstrating significantly weaker affinity towards interfering ions compared to Hg^2+^ ions.

The process of regeneration is essential for the development of affordable and efficient adsorbents. The findings indicated that the Fe_2_O_3_/SiO_2_–NH_2_ magnetic nanocomposite exhibits a diminished adsorption capacity under conditions of low pH. Hence, employing acid treatment could be a suitable method for the restoration of nanocomposites. The adsorption–desorption experiment involved introducing the adsorbent, which had previously adsorbed Hg^2+^ ions, into 25 mL of a solution containing 0.1 M HNO_3_ and 0.1 M HCl and keeping it at a constant temperature of 25 °C for a duration of 12 h. Subsequently, the adsorbent underwent three rounds of washing with distilled water, followed by magnetic separation from the solution. Figure 13c illustrates the adsorption efficiency of the Fe_2_O_3_/SiO_2_–NH_2_ nanocomposite over five consecutive cycles of the Hg^2+^ ions adsorption–desorption process. The findings indicate a decrease in the ability of the nanocomposite to adsorb ions over multiple cycles. The activity had a decline from about 92.6% to 79.5% over the initial three cycles, then from around 77.2% to 72.8% during the final two cycles. Based on the evidence, it can be inferred that this nanocomposite still holds promise for widespread use in the purification of wastewater at a reasonable cost [82]. The decrease in activity may result from the complete elimination of residual metal ions that were adsorbed into the active sites of the nanocomposite surface by a straightforward washing operation. The presence of Hg^2+^ ions on the surface after each generation cycle can obstruct the active sites, preventing the adsorption of new metal ions [83].

### 3.7. Comparison with Other Adsorbents

The adsorption efficiency of the Fe_2_O_3_/SiO_2_–NH_2_ (1:2) nanocomposite was assessed by comparing its maximum adsorption capacity (q_m_) for Hg^2+^ ions with that of other adsorbents. The findings, presented in Table 11, demonstrate that the nanocomposite has a substantial adsorption capacity of 152.03 mg g^−1^. Moreover, it efficiently eliminates substantial quantities of Hg^2+^ ions, achieving a percentage of 92.6%. These results highlight the significant potential of this innovative adsorbent for effectively removing Hg^2+^ ions from wastewater [84,85,86,87,88,89,90,91,92,93].

### 3.8. Adsorption Mechanism of the Nanocomposite 

**Figure 14:**
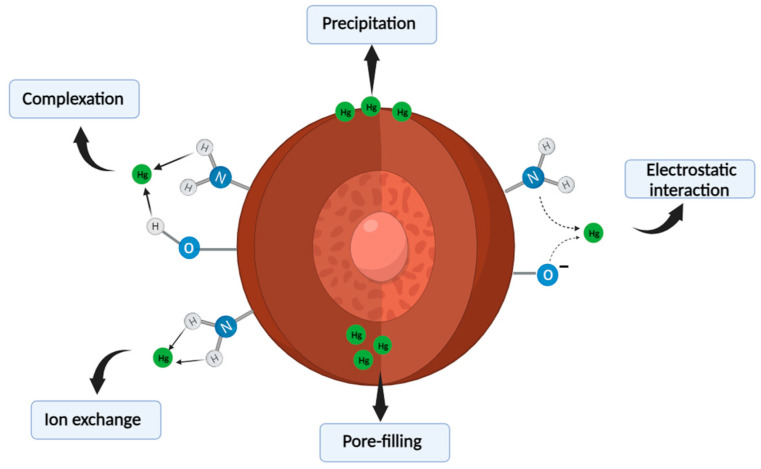
Schematic representation to the interaction’s mechanism using Fe_2_O_3_/SiO_2_–NH_2_ nanocomposite.

Studying the mechanism of the heavy metal adsorption process yields crucial insights into the interactions between the metal ions and composite. This demonstrates the significance of parameters and influential elements in the process of elimination. Many mechanisms are involved in the adsorption of heavy metals, such as complexation, ion exchange, sorption through pores, redox interactions, hydrogen bonding, chemical adsorption, physical adsorption, and electrostatic interactions [94]. The findings from the analysis of the kinetic and adsorption isotherm demonstrate that the kinetic data adhere to PSO, suggesting that the rate-controlling phase of the Hg^2+^ ions adsorption process can be achieved through chemical mechanisms such as complex formation and ion exchange. The adsorption process is influenced by the solution’s pH, which affects the surface charges of the adsorbent and consequently influences their interactions. This can enhance or diminish the adsorption capacity for the removal of Hg^2+^ ions and their interaction with the nanocomposite. Based on the Fourier transform infrared (FT-IR) data shown in Figure 2b, the Fe_2_O_3_/SiO_2_–NH_2_ composite structure has many functional groups, including hydroxy (OH) and amino (NH_2_) groups, which have the ability to gain or lose a proton based on the pH of the solution. In order to clarify the characteristics of the nanocomposite’s surface, the pH at the point of zero charge (pH_pzc_) was measured as shown in Figure 10b. The approximate value is 4.75. When the pH is greater than the pH_pzc_ (4.75), these groups have the ability to release a proton, resulting in a surface that carries a negative charge. As a result, they engage in hydrogen-bond interactions with Hg^2+^ ions, which carry positive charges. Moreover, the adsorbent obtained its greatest removal rate at pH = 5 due to the soft-base nature of its nitrogenous functional groups, which made it more friendly with softer mercury ions. It is evident that the significant peak at 3427 cm^−1^, representing the stretching of -OH and -NH_2_, has shifted to 3441 cm^−1^. This shift indicates the formation of hydrogen-bond-type interactions with the specified metal ions, resulting in their removal from the aqueous solution. In addition, the FT-IR spectra offer compelling evidence of the strong interaction between Hg^2+^ ions and amino groups. The increased intensity of the peak corresponding to -NH bending in Fe_2_O_3_/SiO_2_–NH_2_ (1:2 ratio) after adsorption, compared to Fe_2_O_3_/SiO_2_–NH_2_ (1:2 ratio) before adsorption, can be due to the chemical interaction between Hg^2+^ions and –NH_2_ groups. These functional groups have the ability to eliminate Hg^2+^ ions through complex formation, ion exchange, and hydrogen-bonding mechanisms. An X-ray diffraction (XRD) analysis was performed to confirm the material’s structural integrity before and after adsorption. As depicted in Figure 3b, the primary peaks before and after the following adsorption exhibited similar degrees. However, there were noticeable displacements in the locations of the peaks at 24° and 33°, which corresponded to the (012) and (104) crystallographic planes of α-Fe_2_O_3_ nanoparticles, respectively. The results of our study suggest that the surface of the nanocomposite underwent chemical adsorption of Hg^2+^ ions. Moreover, the crystal structure of the material remains mostly unchanged after the absorption of mercury, demonstrating its exceptional stability. All the data demonstrate that the functional groups have the ability to eliminate Hg^2+^ ions by forming complexes, hydrogen bonding, and engaging in ion exchange pathways, as illustrated in Figure 14.

## 4. Conclusions

The current research involves the synthesis of novel amino-functionalized magnetic Fe_2_O_3_/SiO_2_ nanomaterials with different silicate ratios (1:0.5, 1:1, and 1:2). These materials were utilized as efficient adsorbents for the elimination of Hg^2+^ ions from aqueous solutions. Various techniques were used to characterize the structural, surface, and magnetic properties of the materials. Meanwhile, we examined the conditions under which adsorption occurs, including factors such as pH, the amount of adsorbent used, and the duration of the adsorption process. The optimal pH value was found to be 5.0. The Fe_2_O_3_/SiO_2_–NH_2_ adsorbent with a silicate shell ratio of 1:2 exhibited the maximum capacity for Hg^2+^ ions adsorption, with a value of 152.03 mg g^−1^. This can be due to its notably large specific surface area of 100.1 m^2^ g^−1^, which exceeds other adsorbents. The adsorbent, which has been functionalized with amino groups, has a high affinity for Hg^2+^ ions as a result of the chemical interactions between the metal ions and the amino groups on the surface. The results demonstrated that the PSO model is more effective in describing kinetic behavior compared to other models. The Langmuir isotherm model was determined to be more appropriate for describing the process of adsorption, indicating that a monolayer of Hg^2+^ ions cover the adsorbent surface. Moreover, according to the D–R model, the absorption mechanism of metal ions was predominantly chemical in nature. The analysis of the thermodynamic characteristics revealed that the adsorption of metal ions utilizing magnetic nanocomposite is characterized by spontaneity and endothermicity. The analysis of adsorption–desorption revealed that the adsorbent may exhibit up to five distinct phases, and it can serve as a crucial sorbent in the elimination of heavy metals from water. The present method shows promise and may be regarded as a cost-effective approach for treating wastewater streams.

## Figures and Tables

**Table 1:** EDS compression analysis of synthesized samples and mass percentage of each element.

Sample	Fe (%)	O (%)	Si (%)	N (%)	Hg (%)
Fe_2_O_3_ nanoparticles	34.95	65.05	--	--	--
Fe_2_O_3_/SiO_2_ (1:0.5) ratio	21.81	64.37	13.82	--	--
Fe_2_O_3_/SiO_2_ (1:1) ratio	41.92	42.58	15.50	--	--
Fe_2_O_3_/SiO_2_(1:2) ratio	37.38	41.93	20.69	--	--
Fe_2_O_3_/SiO_2_–NH_2_(1:0.5) ratio	54.49	37.63	7.88	--	--
Fe_2_O_3_/SiO_2_–NH_2_(1:1) ratio	67.34	22.15	8.59	1.92	--
Fe_2_O_3_/SiO_2_–NH_2_(1:2) ratio	54.06	33.47	9.68	2.80	--
Fe_2_O_3_/SiO_2_–NH_2_(1:2) ratio after Hg^2+^ adsorption	33.11	43.78	9.17	2.66	11.28

**Table 2:** The zeta point position values of the prepared samples at pH = 5 ± 0.1 and 30 °C.

Adsorbents	Zeta Potential Value (mV)
Fe_2_O_3_ nanoparticles	−0.85
Fe_2_O_3_/SiO_2_ (1:0.5) ratio	−1.05
Fe_2_O_3_/SiO_2_ (1:1) ratio	−1.36
Fe_2_O_3_/SiO_2_(1:2) ratio	−1.45
Fe_2_O_3_/SiO_2_–NH_2_ (1:0.5) ratio	−1.89
Fe_2_O_3_/SiO_2_–NH_2_ (1:1) ratio	−3.34
Fe_2_O_3_/SiO_2_–NH_2_ (1:2) ratio	−3.50
Fe_2_O_3_/SiO_2_–NH_2_(1:2) ratio after Hg^2+^ adsorption	1.10

**Table 3:** BET surface area values of the prepared samples.

Adsorbents	Specific Surface Area (m^2^ g^−1^)
Fe_2_O_3_ nanoparticles	4.3
Fe_2_O_3_/SiO_2_ (1:0.5) ratio	49.9
Fe_2_O_3_/SiO_2_ (1:1) ratio	68.6
Fe_2_O_3_/SiO_2_ (1:2) ratio	72.4
Fe_2_O_3_/SiO_2_–NH_2_ (1:0.5) ratio	67.9
Fe_2_O_3_/SiO_2_–NH_2_ (1:1) ratio	85.7
Fe_2_O_3_/SiO_2_–NH_2_ (1:2) ratio	100.1

**Table 4:** VSM parameters for Fe_2_O_3_, NPs, Fe_2_O_3_/SiO_2_ (1:2 ratio), and Fe_2_O_3_/SiO_2_–NH_2_ (1:2 ratio) nanocomposite.

Sample	Ms (emu∙g^−1^)	Mr (emu∙g^−1^)	Mr/Ms	Hs (kOe)	Hc (kOe)
Fe_2_O_3_ nanoparticles	1.11	0.211645	0.1906711	1.40	0.26
Fe_2_O_3_/SiO_2_ (1:2)	0.15	0.060153	0.4010187	1.38	0.55
Fe_2_O_3_/SiO_2_–NH_2_ (1:2)	0.14	0.070401	0.5028647	1.36	0.67

**Table 5:** Linear and non-linear equations of pseudo-first-order, pseudo-second-order, and intra-particle diffusion.

Kinetic Models	Equation Form	Equation
Linear pseudo-first-order (PFO)	$\log\left(q_{e}-q_{t}\right)=\log q_{e}-\frac{k_{1}}{2.303}t$	(4)
Non-linear pseudo-first-order (PFO)	$q_{t}=q_{e}(1-e^{-k_{1}t})$	(5)
Linear pseudo-second-order (PSO)	$\frac{t}{q_{t}\;}=\frac{1}{k_{2}{q_{e}}^{2}}+\frac{t}{q_{e}}$	(6)
Non-linear pseudo-second-order (PSO)	$q_{t}=\frac{k_{2}q_{e}^{2}t}{1+k_{2}q_{e}t}$	(7)
Intraparticle diffusion	$q_{t\;=\;K_{p}t^{0.5}\;+\;C}$	(8)

**Table 6:** Kinetics model’s parameters with their correlation coefficient (R^2^) for the adsorption of Hg^2+^ ions on Fe_2_O_3_/SiO_2_–NH_2_ (10 mg) at pH = 5.0 ± 0.1 and 30 °C.

Hg^2+^ Ion Conc. (mg/L)		Pseudo-First-Order			Pseudo-Second-Order			Intraparticle Diffusion			
Linear Form											
	q_e.exp_ (mg g^−1^)	K_1_ (min^−1^)	R^2^	q_e.cal_ (mg g^−1^)	K_2_ × 10^−4^ (g mg^−1^ min^−1^)	R^2^	q_e.cal_ (mg g^−1^)	K_p1_ (mg g^−1^ min^0.5^)	R^2^	K_p2_ (mg g^−1^ min^0.5^)	R^2^
10	51.79	0.11	0.917	66.32	8.82	0.994	52.11	5.76	0.975	0.04	0.813
20	95.20	0.10	0.914	122.0	3.88	0.996	96.24	9.72	0.960	0.10	0.850
30	115.39	0.14	0.924	233.8	2.76	0.990	120.91	15.46	0.989	0.03	0.878
40	131.17	0.11	0.815	215.83	1.96	0.997	141.67	14.21	0.988	0.02	0.852
50	142.93	0.10	0.940	150.5	3.36	0.996	148.71	18.53	0.991	0.04	0.875
**Non-linear form**											
10	51.79	0.032	0.982	56.02	6.29	0.987	54.15	-	-	-	-
20	95.20	0.029	0.994	98.33	2.72	0.996	95.80	-	-	-	-
30	115.39	0.026	0.980	124.37	1.76	0.990	120.34	-	-	-	-
40	131.17	0.024	0.984	138.49	1.54	0.995	132.41	-	-	-	-
50	142.93	0.039	0.960	140.05	1.83	0.980	144.74	-	-	-	-

**Table 7:** Linear and non-linear equations of Langmuir, Freundlich, Tempkin, and Dubinin–Radushkevich.

Isotherm Models	Equation Form	Equation
Linear Langmuir	$\frac{C_{e}}{q_{e}}=\frac{1}{K_{L}\;q_{m}}+\frac{C_{e}}{q_{m}}$	(9)
Non-linear Langmuir	$q_{e}=\frac{q_{m}K_{L}C_{e}}{\left(1+K_{L}C_{e}\right)}$	(10)
Linear Freundlich	$\ln q_{e}=\ln K_{F}+\frac{1}{n}\ln C_{e}$	(11)
Non-linear Freundlich	$q_{e}=K_{F}C_{e}^{1/n}$	(12)
Tempkin	$q_{e}=B\;lnK_{T}+B\;lnC_{e}$	(13)
Dubinin–Radushkevich	$ln\;q_{e}=\ln q_{s}-\beta \varepsilon^{2}$	(14)

**Table 8:** Adsorption isotherms of Hg^2+^ ions on the surface of Fe_2_O_3_/SiO_2_–NH_2_ (10 mg) at 30 °C, pH = 5.0 ± 0.1.

Form	Isotherm	Condition for Applicability	Parameter	Value
Linear form	Langmuir	Monolayer adsorption or homogeneous surface	K_L_ (L mg^−1^)	0.005
			q_max_ (mg g^−1^)	152.03
			R^2^	0.996
Non-linear form			K_L_ (L mg^−1^)	0.730
			q_max_ (mg g^−1^)	146.11
			R^2^	0.956
Linear form	Freundlich	Multi-layers adsorption or non-uniform distribution	1/n	0.285
			K_F_ (mg g^−1^)	63.30
			R^2^	0.844
Non-linear form			1/n	
			K_F_ (mg g^−1^)	82.66
			R^2^	0.914
Linear form	Tempkin	Uniform distribution or heterogeneous surface	B_T_	36.17
			K (L.g^−1^)	11.62
			R^2^	0.930
Non-linear form			B_T_	33.42
			K (L.g^−1^)	21.64
			R^2^	0.940
Linear form	Dubinin–Radushkevich (D–R)	Distinguish between physical and chemical adsorption	q_s_ (mg g^−1^)	129.02
			E (kJ mol^−1^)	9.97
			R^2^	0.957

**Table 9:** Thermodynamic parameters of Hg^2+^ ions adsorption on Fe_2_O_3_/SiO_2_–NH_2_ (1:2) nanocomposite.

Temperature (K)	ΔG° (kJ mol^−1^)	ΔH° (kJ mol^−1^)	ΔS° (J mol^−1^ K^−1^)
298	−50.64	41.84	170.22
303	−51.57		
308	−52.42		
313	−53.28		
318	−54.13		

**Table 10:** Results of distribution coefficient (K_d_) and selectivity coefficient (K_s_) of Fe_2_O_3_/SiO_2_–NH_2_ toward different metal ions.

Metal Ion	Before Adsorption (C_o_) (mgL^−1^)	After Adsorption (C_e_) (mgL^−1^)	Adsorption Efficiency (%)	K_d_ (mLg^−1^)	K_s_
Mg^2+^	20.05	19.64	2.04	104.4	437.3
Co^2+^	20.09	19.21	4.38	229.0	199.4
Ni^2+^	20.14	18.76	6.85	367.8	124.1
Cu^2+^	20.05	18.62	7.13	383.9	118.9
Cd^2+^	20.07	17.65	12.06	685.6	66.6
Hg^2+^	20.06	1.98	90.13	45,656.5	--

**Table 11:** Evaluation of present Hg^2+^ ion adsorption characteristics on Fe_2_O_3_/SiO_2_–NH_2_ (1:2) nanocomposite in comparison to those of various adsorbents reported in the literature.

Adsorbents	pH	Adsorption Capacity (mg g^−1^)	Reference
Magnetic mesoporous silica nanospheres	4	303.03	[83]
Cysteine–carbon/Fe_3_O_4_	2	94.33	[84]
Di hydrolipoic acid/Fe_3_O_4_	7	140.8	[85]
Fe_3_O_4_/SiO_2_/selenium	3	70.42	[86]
Fe_3_O_4_/SiO_2_–NH–COOH	5	72.30	[87]
CoFe_2_O_4_/SiO_2_–EDTA	7	103.3	[88]
Fe_3_O_4_-graphite nanosheets/NH_2_	6	96.15	[89]
Fe_3_O_4_/Au	9	79.59	[90]
Ethylene glycol bis thioglycolate–Au/Mn–Fe_3_O_4_ NPs	5.5	23.10	[91]
Curcumin-based magnetic nanocomposite	6	144.9	[92]
Fe_2_O_3_/SiO_2_–NH_2_ nanocomposite with silicate ratio (1:2)	5	152.03	This work

## Data Availability

The raw data supporting the conclusions of this article will be made available by the corresponding author upon request.
